# Growing pains: a scoping literature review of how perinatal psychiatry was impacted by COVID-19

**DOI:** 10.1192/bjo.2021.615

**Published:** 2021-06-18

**Authors:** Stephanie Adeyemi

**Affiliations:** East and North Hertfordshire NHS Trust

## Abstract

**Aims:**

This scoping review aims to assess the impact of COVID-19 on the field of Perinatal Psychiatry and identify any innovations made as a result of this.

**Background:**

The World Health Organisation declared the COVID-19 outbreak a global pandemic on March 11th 2020. This pandemic has transformed the way in which Perinatal Psychiatric services are delivered in the United Kingdom and countries across the globe acting as a catalyst for innovation.

**Method:**

The databases searched for peer reviewed literature written since December 2019 were: PsychINFO, MEDLINE, EMBASE, CINAHL and PUBMED. Search strategy key words were: COVID-19, SARS-CoV-2, perinatal psychiatry and maternal mental health. Arksey and O'Malley's framework was utilised. Data were collated and summarized thematically.

**Result:**

42 studies met the inclusion criteria. The aforementioned studies included data from over 60,000 women from the following countries: China, Italy, Netherlands, United States, United Kingdom, Brazil, India, Spain, Ireland, Norway, Switzerland, Iran, Japan and Nepal. Literature clearly indicates that during the pandemic there was an increase in depression and anxiety. Risk factors included: financial insecurity, disrupted antenatal care, isolation, poor physical health and domestic violence. Evidence also suggested COVID-19 stressors impacted feeding practices and infant development as cytokines pass from mother to baby.

Perinatal Psychiatry services have adopted social media apps to provide antenatal information, teleconsultations, smartphone-based cognitive-behavioral therapy (iCBT) programs and increased utilisation of screening tools such as the Pandemic-Related Pregnancy Stress Scale (PREPS), the Edinburgh Postnatal Depression Scale (EPDS) and the Postpartum Specific Anxiety Scale.

**Conclusion:**

Whilst this review features literature centred on women from across the globe African women are underrepresented. This should be addressed in future studies. This review shows that the COVID-19 pandemic has impacted maternal mental health and acted as a catalyst for innovation. It is essential that efforts are made to support women during pregnancy and the perinatal period now more than ever.

